# Enhancing High-Alloy Steel Cutting with Abrasive Water Injection Jet (AWIJ) Technology: An Approach Using the Response Surface Methodology (RSM)

**DOI:** 10.3390/ma17164020

**Published:** 2024-08-13

**Authors:** Andrzej Perec, Elzbieta Kawecka, Frank Pude

**Affiliations:** 1 Faculty of Technology, Jacob of Paradies University, 66-400 Gorzów Wielkopolski, Poland; ekawecka@ajp.edu.pl; 2 Inspire AG (ETH Zurich), CH-8005 Zurich, Switzerland; frank.pude@inspire.ch; 3 Steinbeis Consulting Center High-Pressure Waterjet Technology, 86497 Horgau, Germany

**Keywords:** abrasive water injection jet, surface roughness, high-alloy steel, response surface method, modeling

## Abstract

The common machining technologies for difficult-to-machine materials do not remarkably ensure acceptable efficiency and precision in bulk materials cutting. High-energy abrasive water injection jet (AWIJ) treatment can cut diverse materials, even multi-layer composites characterized by divergent properties, accurately cutting complex profiles and carrying them out in special circumstances, such as underwater locations or explosion hazard areas. This work reports research on the AWIJ machining quality performance of X22CrMoV12-1 high-alloy steel. The response surface method (RSM) was utilized in modeling. The most influencing process control parameters on cut kerf surface roughness—abrasive flow rate, pressure, and traverse speed—were tested. The result is a mathematical model of the process in the form of a three-variable polynomial. The key control parameter affecting the cut slot roughness turned out to be the traverse speed. In contrast, pressure has a less significant effect, and the abrasive mass flow rate has the slightest impact on the cut slot roughness. Under the optimal conditions determined as a result of the tests, the roughness of the intersection surface *Sq* does not exceed 2.3 μm. Based on the ANOVA, we confirmed that the model fits over 96% appropriately with the research outcomes. This method reduces the computations and sharply determines the optimum set of control parameters.

## 1. Introduction

Abrasive water injection jet cutting (AWIJ) is a non-conventional machining process that relies on simultaneous material removal caused by high-pressure water jet erosion and erosion caused by abrasive grains. The high-velocity jet, generated in a high-pressure pump and nozzle, removes the loose abrasive grains and then is concentrated in the focusing tube. The material removal takes place by the total results of microplastic deformation, micro-cutting erosion by the abrasive grains in the high-energy jet, and crack formation and proliferation.

Processing by AWIJ is characterized by great versatility, resulting from the option of processing a variety of materials, including rocks [1], ceramics and glass [2], hard metals [3,4], composites [5,6], special structural 3D materials [7], heavy-to-machine metals [8,9], superalloys [10], human bone [11], bone cement [12], and even food products [13]. It is suitable for creating complex contours and cutting thin materials. Another, no less important advantage is the lack of a heat-affected zone. Unlike most traditional cutting methods, AWIJ generates a minimal amount of heat in the cutting operation, and the temperature of the cutting area does not exceed 62 °C [14]. For this reason, it is also suitable for cutting heat-sensitive materials, such as plastics and foams. Due to the lack of heat influence, material deformations are negligible, and thermal damage is eliminated.

AWIJ ensures fine tolerances (usually within 0.1 mm) and excellent cut surface quality [15]. It is perfect for precision parts [16] and complex designs, and thanks to the narrow cutting kerf, it causes less material loss compared to other cutting methods. This material separation technique is also environmentally friendly; AWIJ does not produce hazardous fumes and used water and abrasive material can be recycled [17,18]. 

The properties of abrasives [19,20], particularly geometric features, are key in AWIJ operations for realizing good efficiency and, of course, precision [21] on the machined surfaces.

Material treatment with AWIJ, like laser processing [22], is an advanced manufacturing technology. It is more complex than typical machining and hence modeling [23] and optimization [24] were applied to reach better treatment effects. Modeling and optimization with different approaches were utilized in other areas; for example, in surface treatment [25,26], some chemical [27] or epoxidation processes [28,29], cutting of human bone [30] or animal bone [31], and even in digital signal processing [32].

Research on the processing of different steel grades using AWIJ is the focus of research in diverse research institutions.

The goal of Sisodia et al. [33] was to examine how the factors of the AWIJ machining process affect the attributes of the kerf taper and surface roughness of quenched AISI 440C steel. The predictive model that has been developed incorporates alike coded and uncoded outputs. The analysis reveals that the model’s projected values correspond with the tested parameters. 

In the case of the D2 steel drilling process, Mahalingam et al. [34] proposed using the harmony search algorithm (HSA), a novel metaheuristic method, to determine the optimal value of the selected control factors of AWIJ, such as jet pressure, stand-off distance, and abrasive expenditure, under the modern minimization of the selected bored hole assets because the surface errors and shape errors present very well fit of the raw data to the regression line.

Research on how the steel structure and heat treatment affect AWIJ machining efficiency was reported by Hlavacova et al. [35]. Various heat treatment techniques were used for three distinct steels, medium-carbon steel C45, alloyed heat-treated steel 37MnSi5, and alloy special steel 30CrV9. These treatments included normalizing annealing, soft annealing, quenching, and quenching with tempering. After that, it was sliced using the same cutting parameters by an AWIJ. It examined the correlations among the surface roughness metrics *Ra*, *Rz*, and root mean square (*RMS*) and the mechanical properties of heat-treated steels. The most significant determinant of cutting quality appeared to be the homogeneity of the steel microstructure; the more the roughness values varied with the hardness of the structural elements in the heterogeneous structure.

Miao et al. [36] demonstrated how the primary process factors affect cutting depth and developed a simulation model using the SPH-coupled FEM approach to replicate the erosion phenomenon of an abrasive water jet. Additionally, the authors introduced the typical parametric model of removal volume. The findings demonstrate that the parametric model may be used to base the setup of control factors and anticipate the cutting depth of AISI 304.

The machining of EN31 steel using an AWIJ was shown by Kant et al. [37]. The study examined the effects of pressure, abrasive expenditure, stand-off distance, and traverse speed as control factors on the machining time and surface roughness. Every parameter that was assigned was optimized to have the least amount of surface roughness and the shortest treatment time possible using Grey Relational Analysis.

Research on AWIJ treatment of various metals, involving a high-chromium tool D3, was reported by Akkurt [38,39]. The results of material sort and thickness on machining time were examined and considered. The hardness study’s findings showed that there is little correlation between the material’s hardness and its machined surface. The material’s mechanical characteristics and microstructure are unaffected by machining.

Arun et al. [40] made a novel attempt to search the impact of different control parameters of the AWIJ machining process over the treatment of ferritic–austenitic stainless steel 2205. The work was mainly concentrated on obtaining a lower angle of the cut slot and minimal surface roughness. The Taguchi L9 orthogonal array was employed for the response optimization noted after the treatment. The effects established that the stand-off distance is the most affecting control factor, afterward is traverse speed, and the smallest influence is the abrasive expenditure.

Doreswamy et al. [41] described the process of the influence of feed rate, stand of distance, and *Ra* roughness parameter on the slot surface during the treatment of stainless steel tool D2 using an AWIJ. The outcomes also confirmed that the surface roughness (*Ra*) value increases as the stand-off distance and feed rate increase.

To understand and estimate AWIJ treatment of various steels, with the 1.7131 high-grade structural steel, Hlavac introduced an analytical model [42]. This model demonstrated the potential of AWIJ for developing steering software with increased computation rates and accuracy in machining effects determination.

Prazmo et al. [43] reported the phenomena of abrasive grain disintegration in the succeeding stages of high-velocity abrasive water jet generation and the material treatment of this AWIJ. They looked at garnet fracture in AWIJ cutting both during AWIJ’s creation and the whole cutting process. The abrasive’s erosion efficiency was assessed using a study of the recovered abrasive following treatment. The potential for reusing abrasive materials and the financial implications of this process were also thoroughly examined.

To identify the appropriate abrasive material for machining ductile materials with AWIJ, Gent et al. [44,45] presented tests on the impacts of some mineral abrasives and high-density glass. The authors demonstrated that this kind of erosion rate does not improve above a specific abrasive density and that polycrystalline abrasives outperform monocrystalline abrasives with the same composition.

The analysis of the state of the art confirmed that the treatment of high-alloy steels is feasible with AWIJ. It is a very effective tool for machining high-alloy steel due to its cold working properties and huge machining capabilities. In other studies, the authors dealt with cutting various grades of steel, from structural to tool steel, using AWIJ. However, in most cases, the efficiency of the cutting process was analyzed, not its quality. In a few cases, the authors assessed the roughness of the cut surface using *Ra* or *Rz* parameters, which determine the roughness in only one profile. Previous research with such materials was presented only in the range of drilling and comparison of erosion rates. Hence, this article introduces a new investigation on the accuracy of cutting difficult-to-machine 1.4923/X22CrMoV12-1 high-alloy steel, and in this range, the article performs scientific innovation.

## 2. Materials and Methods

### 2.1. Target Material

High-alloy stainless steel 1.4923/X22CrMoV12-1, categorized as creep-resisting steel, is a grade with a martensitic structure intended for parts, subassemblies, and blades and rotor components for steam turbines that are forged at temperatures as high as 600 °C. This steel is often used for structures with high resistance to fatigue stresses. Products of the 1.4923/X22CrMoV12-1 grade are also used to make components and parts of airplane structures, parts for devices for the needs of the chemical, oil and gas industries, and likewise, as components for energy engineering. The 1.4923/X22CrMoV12-1 grade belongs to difficult-to-weld steels. Its chemical compositions are present in Table 1.

Creep-resisting steel 1.4923/X22CrMoV12-1 is a very popular material for turbine, valve, and pump producers. According to the EN 10269 norm [46], this steel is produced in two variants, QT1 and QT2 (Table 2). The biggest difference is in mechanical properties. QT2 does not conform with the PED 2014/68EU requirements for KV min. 27 J.

### 2.2. Abrasive Material

The almandine garnet was applied as an abrasive material in the tests. Garnets are classified as silicate minerals with related crystal structures, however various the chemical compositions. Almandine is the most famous and well-known member of the garnet group. Its most characteristic properties are presented in Table 3. 

**Figure 1:**
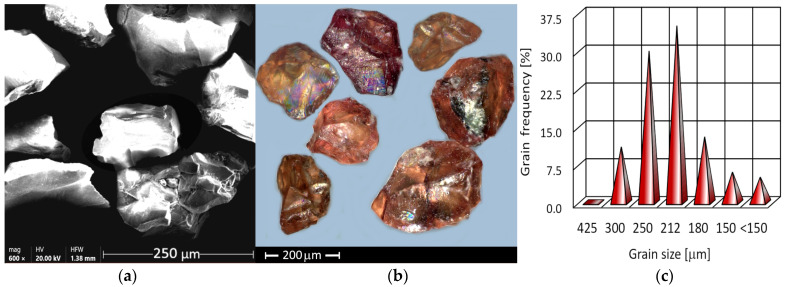
J80A garnet: (**a**) grain SEM image, (**b**) light microscope image, (**c**) particle distribution.

J80A almandine garnet comes from the Jiangsu Province ledge in China and is made of crushed rock. The form of the grains is near isometric. Additionally, its grains are described by sharp edges and angles (Figure 1a,b), which positively affect the efficiency of AWIJ machining. The grain size distribution of the J80A garnet is presented in Figure 1c. The grain distribution is close to normal with a predominance of grains ranging in size from 212 to 250 μm, constituting over 60% of the total population.

### 2.3. Test Rig and Cutting Method

Cutting investigations were conducted on a test rig equipped with an OMAX 60120 water jet cutting machine, manufactured by OMAX Corp, Kent, WA, USA. They involved cutting the tested material by directing the AWIJ perpendicularly and causing it to move [47,48] at a precisely defined speed. The cut by AWIJ was conducted under the following conditions:

- Pressure: 360–400 MPa;
- Traverse speed: 50–250 mm/min;
- Abrasive flow: 250–450 g/min;
- Abrasive almandine garnet 80 Mesh;
- Water nozzle internal diameter: 0.30 mm;
- Focusing tube internal diameter: 0.76 mm;
- Stand-off distance: 4 mm.

### 2.4. Response Surface Methodology (RSM)

RSM is a combination of mathematical and statistical modeling techniques. It may also be applied to optimization using several criteria. Furthermore, it guarantees a relationship between process control parameters and perceived reactions. The polynomial equation of three variables (Equation (1)) determines a regression model.

(1) $$y=\beta_{0}+\sum_{i=1}^{k}\beta_{i}x_{i}+\sum_{i=1}^{k}\beta_{ii}x_{i}^{2}+\varepsilon$$

where 

*y* is the dependent factor; 

*x*_i_ is the value of the *i*-th control factor; 

*k* is the number of control factors; 

*β*_0_, *β_i_*, *β_ii_* are the coefficients of regressions; 

*ε* is the error.

### 2.5. Cut Kerf Roughness

**Figure 2:**
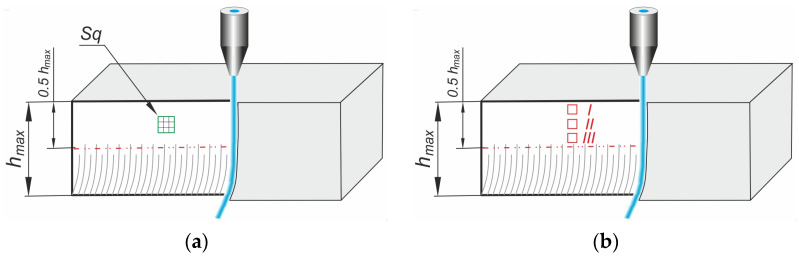
Measurements and observation areas on (**a**) optical microscope, (**b**) SEM microscope.

Cut surface roughness measurements were conducted on the area of about 2.85 mm × 2.85 mm at stitching mode (elemental area 0.95 mm × 0.95 mm) on an Olympus DSX1000 3D microscope, manufactured by Olympus Corp., Tokyo, Japan. The measurement area was chosen in the center of the useful cutting depth region. The useful cutting depth is approximately half the maximum depth *h_max_* (Figure 2a).

The surface detail was observed on a ThermoFisher Scientific SEM microscope, Axia ChemiSEM, manufactured by Thermo Fisher Scientific Corp., Waltham, MA, USA, running at 10 keV accelerating voltage in the low-vacuum condition. The observation area was chosen in the top (I), middle (II), and bottom (III) of the effective cutting depth zone.

**Figure 3:**
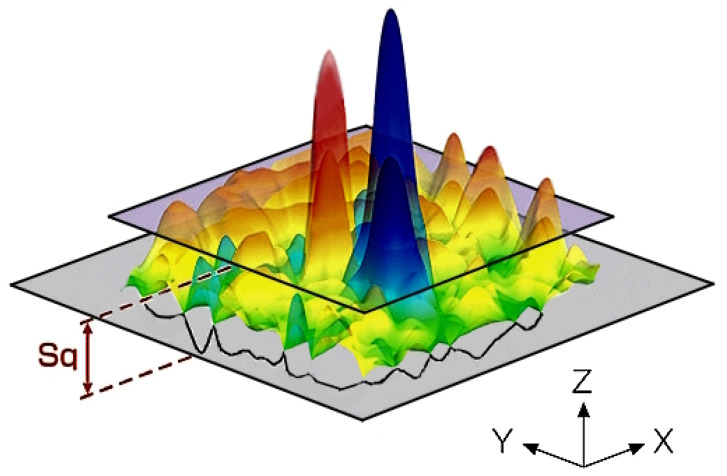
Illustration of the *Sq* surface roughness factor.

Root mean square height (*Sq*) was chosen as the measure of roughness. This parameter extends the contour (line of roughness) parameter *Rq* to 3D. It represents the root mean square for *Z*(*x*, *y*) within the evaluation zone (Figure 3). The *Sq* factor is a universal measure of the texturing surface and is insensible in differentiating tops, valleys, and the various texture spacing properties. The following equation (Equation (2)) defines this roughness factor:

(2) $$Sq=\sqrt{\frac{1}{A}\iint_{A}Z^{2}\left(x,\;y\right)dxdy}$$

It is also known as the RMS value and is one of the most utilized parameters. The height distribution’s standard deviation is matched by the *Sq* parameter. Since the parameter is not greatly impacted by scratches, pollution, or measurement noise, it generates good statistics and allows for consistent findings.

## 3. Results and Discussion

The analysis of the variance of the test effects is given in Table 4. Its analysis was conducted with a 95% confidence level (α = 0.05). The *p*-value < 0.05 suggests that this model factor is statistically significant. To test multicollinearity, the variance inflation factor (VIF) was taken into consideration. It measures how strong multicollinearity is. The model’s multicollinearity contributes to an inflated variance of the assessed regression component, which is shown by the VIF. Multicollinearity does not exist when VIF is 1.0. No multicollinearity was seen for any of the components that were examined because for each factor the VIF = 1.00 (Table 4).

Based on the coefficients shown in Table 4, the final surface roughness control model (Equation (3)) was formulated:

(3) $$S_{q}=-125.3+0.0491r_{A}+0.604p+0.0517v_{p}-0.00006r_{A}^{2}-0.0008p^{2}+\;-0.00002v_{p}^{2}-0.0001r_{A}\cdot p+0.000002r_{A}\cdot v_{p}-0.000093p{\cdot v}_{p}$$

where

*S_q_* is the roughness factor [μm];

*P* is pressure [MPa]; 

*v_p_* is traverse speed [mm/s]; 

*r_A_* is flow rate [g/s].

An R^2^ calculation was used to assess the model’s performance. A statistical indicator of how closely the regression line resembles the actual data points in regression is the R^2^ coefficient of determination. The regression standard error S = 0.260825, R^2^ over 96%, and R_adj_^2^ over 94% are presented in Table 5. The R^2^ values near 95% indicate a good enough match with the raw data for the regression line.

**Figure 4:**
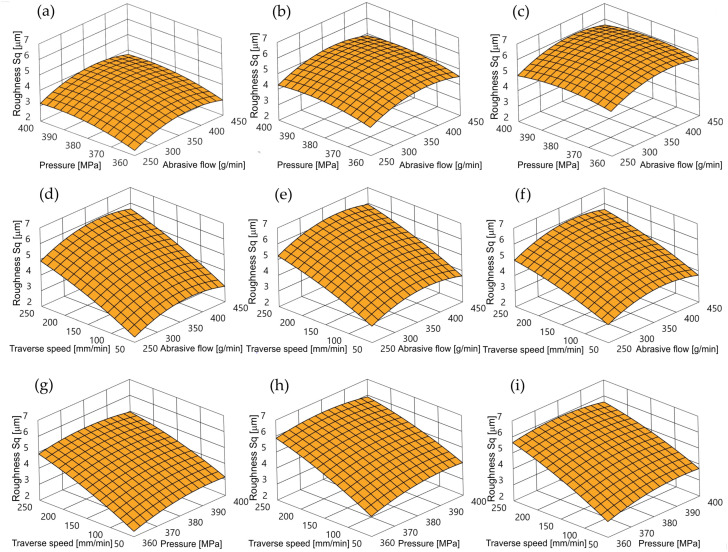
Impact of control factors on *Sq* surface roughness: (**a**) traverse speed 50 mm/min, (**b**) traverse speed 150 mm/min, (**c**) traverse speed 250 mm/min, (**d**) pressure 360 MPa, (**e**) pressure 380 MPa, (**f**) pressure 400 MPa, (**g**) abrasive flow 250 g/min, (**h**) abrasive flow 350 g/min, (**i**) abrasive flow 450 g/min.

A graphic illustration of Equation (3) is presented in Figure 4. The pressure modification has no significant impact on the *Sq* roughness factor. Only a slight increase in roughness proportional to the increase in pressure can be observed here. This is due to a certain increase in the kinetic energy of the abrasive grains. Having greater potential, abrasive grains perform deeper micro-cutting, which increases roughness.

The bigger influence is the traverse speed. The increase of the traverse speed causes an increase in the *Sq* factor. This is primarily due to the larger number of grains capable of micromachining in the cutting area per time unit. 

In the case of the influence of the abrasive flow rate, a reduction in the *Sq* roughness coefficient was observed both when the highest and the lowest abrasive flow rates were used. 

To ensure technological efficiency in the conditions of achieving low machined roughness, it is necessary to maintain low feed and low pressure. However, the requirements to maintain high economic efficiency suggest setting the abrasive flow rate as low as possible to achieve the low roughness of the cut surface.

**Figure 5:**
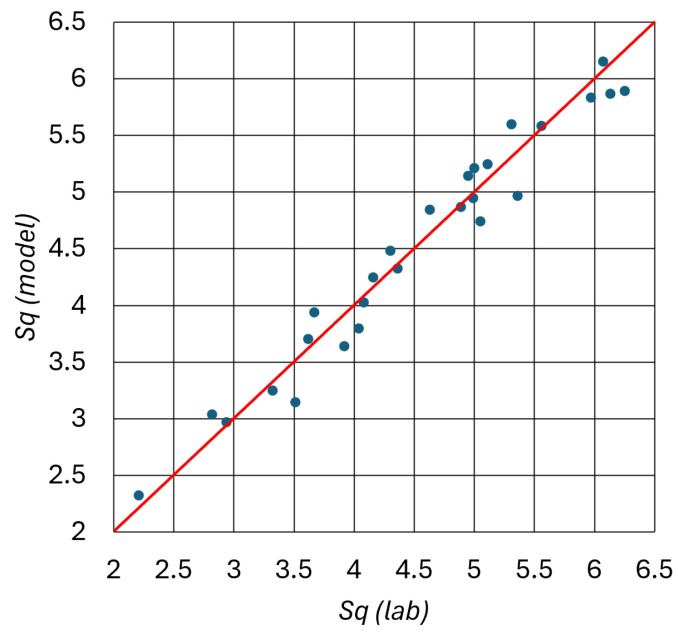
Scattering graph of the modeled and measured *Sq* surface roughness.

The raw data fit well with the regression line and the scattering plot (Figure 5), which confirms this accordance because the points are relatively near to the red line. It implies that the established polynomial equation of the high-alloy steel surface roughness process is satisfactory.

**Figure 6:**
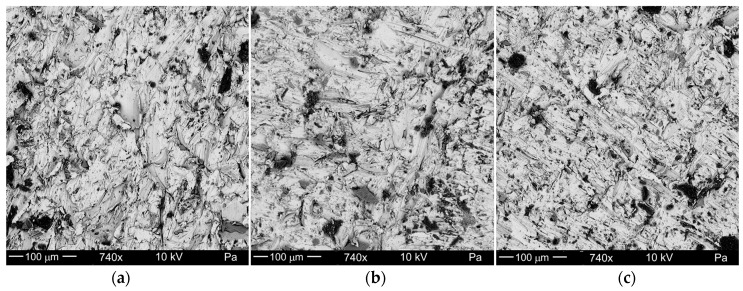
Example view of cut surface roughness of high-alloy steel: (**a**) top area I, (**b**) middle area II, (**c**) bottom area III.

Exemplary SEM images of the cut surface (according to the schematic view in Figure 2b) are presented in Figure 6. Shallow erosion traces become deeper in the lower part of the material. Parallel, shallow traces of micro-cutting (Figure 6a) are visible in the sample’s upper part (I) and pass smoothly in more chaotic machining traces (Figure 6b) in the middle part (II) of the sample.

In the lower zone (III) (Figure 6c), the machining marks become even more chaotic and sometimes randomly crossed. The most characteristic traces of erosion of high-alloy steel left by AWIJ are shown in Figure 6a. Here, they are able to be seen in the structure of parallel traces.

## 4. Conclusions

With AWIJ machining, the RSM approach worked well for optimizing the cutting control parameters. This technique finds the ideal combination of control settings with a sharp reduction in calculations. The RSM approach can also resolve issues with contradictory answers. For the selected optimal parameters, low surface roughness parameters were obtained. The following findings were drawn from the tests on the modeling of 1.4923/X22CrMoV12-1 high-alloy steel cutting that was performed:

- Traverse speed has a significant impact on surface roughness;
- Abrasive flow has a second important impact on surface roughness;
- In the whole investigation scope of control factors, the best roughness of the cut surface was noticed for traverse speed near 50 mm/min, 250 g/min abrasive flow, and 400 MPa pressure;
- The R^2^-(squared) factor indicates how well the model fits the experimental data; it represents the proportion of response variance described by the model, and it is over 96.1% in this case;
- A good model fit is also confirmed by the R_adj_^2^ value of 94.03%. R_adj_^2^ is adjusted for the ratio of the number of predictors in the model to the number of tests;
- Multicollinearity was not detected for the model’s regression coefficients.

To obtain low machined roughness, the lowest possible feed rate, low pressure, and the lowest abrasive flow should be used. However, it should be kept in mind that achieving high machining efficiency may require the use of a different level of control parameters.

In further research, the process of cutting other materials by AWIJ will be modeled and optimized using the RSM technique. Included will also be the impact of additional control factors on the AWIJ cutting process.

## Figures and Tables

**Table 1:** Chemical composition of 1.4923/X22CrMoV12-1 high-alloy steel.

Content	Cr	Mo	W	Mn	Ni	V	C	Si	S	P
Min. [%]	11.0	0.8	<0.6	0.30	0.3	0.25	0.20	0.10	<0.03	<0.03
Max. [%]	12.5	1.2		0.80	0.80	0.35	0.26	0.50		

**Table 2:** Mechanical properties of 23H12MNF high-alloy steel.

QT Variant	0.2% Proof Strength R_p0.2_ [MPa]	Tensile Strength R_m_ [MPa]	Impact Energy KV [J]
QT1	600	800–950	27
QT2	700	900–1050	20

**Table 3:** Properties of almandine garnet.

Characteristic	Description
Mineral Composition	The chemical formula is Fe_3_Al_2_(SiO_4_)_3_.
Color	Almandine is typically deep red, brownish-red, or purplish red. Its red hue is caused by the presence of iron in its chemical structure.
Hardness	On the Mohs scale of mineral hardness, garnets, including almandine, have a hardness of 6.5 to 7.5. This makes them durable gemstones, suitable for use in jewelry.
Transparency	Almandine is usually transparent to translucent, which means light can pass through it, but it might not be clear.
Luster	It has a vitreous to resinous luster when polished.
Crystal Structure	Almandine garnets belong to the isometric crystal system. They typically form dodecahedra or trapezohedron.
Occurrence	Garnets, including almandine, can be found in metamorphic and igneous rocks. They are often found in association with minerals like mica, feldspar, and quartz.
Uses	Almandine garnets are popular abrasive materials for sandpaper and especially for abrasive water jet cutting due to their unique composition of properties like hardness, density, and grain shape.

**Table 4:** Surface roughness model summary.

Source	DF	Adj SS	Adj MS	F-Value	*p*-Value	VIF
Model	9	28.461	3.1623	46.48	46.48	
Linear	3	25.061	8.3536	122.79	122.79	
Flow rate	1	1.9405	1.9405	28.52	28.52	1.00
Pressure	1	0.7200	0.7200	10.58	10.58	1.00
Traverse speed	1	22.400	22.400	329.27	329.27	1.00
Square	3	2.9721	0.9907	14.56	14.56	
Flow rate × Flow rate	1	2.1720	2.1720	31.93	31.93	1.00
Pressure × Pressure	1	0.5521	0.5521	8.12	8.12	1.00
Traverse speed × Traverse speed	1	0.2481	0.2481	3.65	3.65	1.00
Two-Way Interaction	3	0.4275	0.1425	2.09	2.09	
Flow rate × Pressure	1	0.0056	0.0056	0.08	0.08	1.00
Flow rate × Traverse speed	1	0.0075	0.0075	0.11	0.11	1.00
Pressure × Traverse speed	1	0.4144	0.4144	6.09	6.09	1.00
Error	17	1.1565	0.0680			
Total	26	29.617				

DF, degree of freedom; SS, sum of squares; MS, mean square; F, ratio of variance error; VIF, variance inflation factor.

**Table 5:** Surface roughness model summary.

S	R^2^	R_(adj)_^2^	R_(pred)_^2^
0.260825	96.10%	94.03%	89.94%

## Data Availability

No new data were created or analyzed in this study. Data sharing is not applicable to this article.
